# TilapiaVisionDataset: Annotated image dataset for oreochromis niloticus

**DOI:** 10.1016/j.dib.2026.113182

**Published:** 2026-08-21

**Authors:** Adriano Carvalho Costa, Elias Marques de Oliveira, Alessa Pereira Diniz Araújo Araújo, Brenno Muller Vitorino, Vitória de Vasconcelos Kretschmer, Gabriela Almeida Marques, Lessandro do Carmo Lima, Gidelia Araujo Ferreira de Melo, Heyde Francielle do Carmo França, Sergio Souza Novak

**Affiliations:** Instituto Federal Goiano, Rodovia Sul Goiana, Km 01, Zona Rural, Rio Verde, Goiás, 75901-970. Brazil

**Keywords:** Precision aquaculture, Automated fish counting, Computer vision, Deep learning, YOLO format, Flume monitoring

## Abstract

Precision aquaculture requires automated monitoring systems capable of supporting fish management and production control in intensive farming environments. This article describes the TilapiaVisionDataset [3], a curated image dataset developed for object detection and counting of Nile tilapia (Oreochromis niloticus) under controlled aquaculture conditions. The dataset was generated using an experimental water flume designed to simulate fish handling and monitoring scenarios, operating with a controlled water flow rate of 2700 L/h to reproduce realistic movement and interaction patterns while maintaining a low-stress acquisition procedure. Image acquisition was performed using a fixed high-resolution camera positioned above the flume, resulting in 8512 images with a spatial resolution of 1920 × 1080 pixels. A total of 63,004 fish instances were manually annotated using bounding boxes following the YOLO annotation format. The dataset includes scenes characterized by high stocking density, averaging 7.40 fish per frame, and incorporates common visual challenges observed in aquaculture environments, such as fish overlap, partial occlusion, motion variability, reflections, and illumination changes. To support machine learning workflows, the dataset is organized into training (70%), validation (15%), and testing (15%) subsets. The annotation structure and dataset organization enable direct compatibility with modern computer vision frameworks and object detection architectures, including single-stage and two-stage deep learning models. The dataset can be reused for the development, benchmarking, and validation of automated fish detection, counting, and monitoring systems. Potential applications include algorithm training, comparative evaluation of detection models, robustness analysis under dense biological scenarios, and research involving computer vision techniques applied to aquaculture monitoring environments.

Specifications Table

**Table:** 

Subject	Biology
Specific subject area	Aquaculture and Computer vision applied to automated fish detection, counting, and monitoring in precision aquaculture systems.
Type of data	Type of data: Image; Annotated dataset. Data format: Raw; Annotated; Processed (JPG image files and TXT annotation files in YOLO format).
Data collection	Images were collected using a Logitech C920 Full HD camera (1920×1080 px, 30 fps) positioned 1.20 m above a PVC flume operating with controlled water flow (2700 L/h). Video recordings were obtained under controlled lighting and converted into image frames. Frames containing visible Nile tilapia individuals were selected, while blurred or empty frames were excluded. Fish were manually annotated using bounding boxes in YOLO format, ensuring consistent labeling and dataset normalization for training and evaluation tasks.
Data source location	Institution: Instituto Federal Goiano, Campus Rio Verde City: Rio Verde, Goiás Country: Brazil Latitude/Longitude: −17.8311, −50.9408
Data accessibility	Repository name: Mendeley Data Data identification number (DOI): 10.17,632/bnst7jsdcx.1 Direct URL to data: https://data.mendeley.com/datasets/bnst7jsdcx/1 Instructions for accessing these data: The dataset is publicly available and can be accessed directly through the repository link without restrictions or special permissions.
Related research article	*None.*

## 1 Value of the Data

• The TilapiaVisionDataset contains 8512 annotated images with 63,004 Tilapia instances, providing a high-density dataset suitable for training and benchmarking deep learning models for automated fish detection and counting in aquaculture.
• The dataset supports computer vision applications such as non-invasive environmental monitoring, automated fish counting, spatial distribution analysis, behavioral monitoring, and ecological assessment in production and natural aquatic environments.
• The data can be integrated into camera trap systems and Edge AI embedded devices, and its YOLO annotation format enables the development of real-time identification and monitoring solutions for precision aquaculture.
• Validation experiments using YOLO-based detectors and Faster R-CNN architectures achieved high mAP@0.5 performance, supporting the dataset’s use as a benchmark for evaluating convolutional neural networks (CNNs) in complex aquatic scenarios.

## 2 Background

Recent advances in aquaculture have promoted the integration of artificial intelligence and computer vision techniques to support automated monitoring and management of fish production systems [1]. Image-based approaches enable non-invasive data acquisition, reducing handling stress while allowing continuous observation during farming operations. The development of reliable deep learning models for fish detection and counting depends on the availability of structured and annotated datasets representing realistic production conditions [2].

**Fig. 1:**
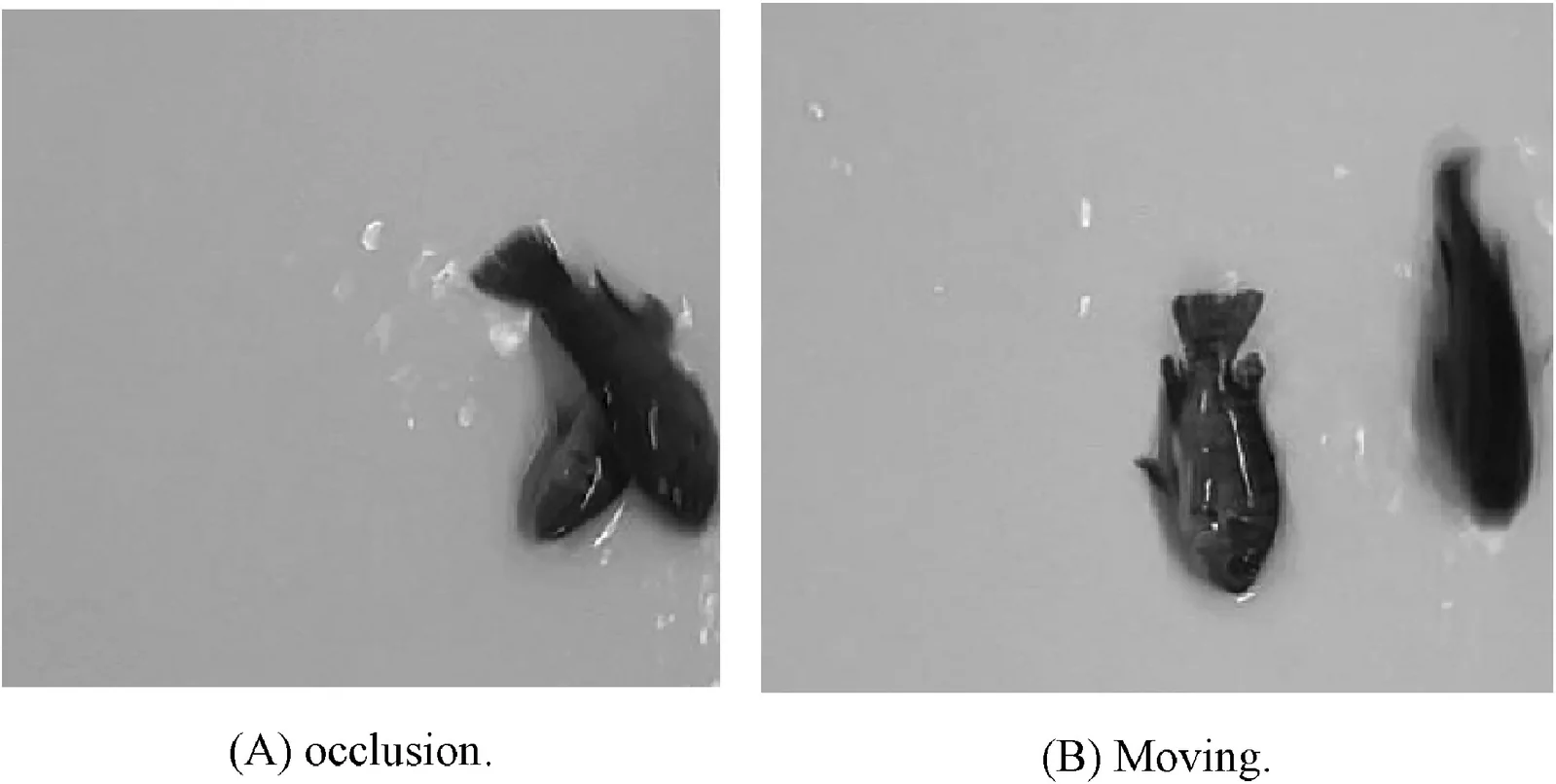
Object occlusion and overlapping and Object movement and lighting variations.

The TilapiaVisionDataset [3] was developed to support the training and validation of automated Nile tilapia detection and counting methods. The dataset contains images acquired in an experimental flume designed to simulate aquaculture handling environments, capturing fish movement under controlled water flow conditions. Data collection focused on representing practical challenges commonly observed in aquatic monitoring scenarios, including fish overlap, motion variability, lighting fluctuations, and surface reflections [3]. As shown in Fig. 1, object occlusion and overlapping, as well as object movement and lighting variations, can significantly affect detection performance.

The dataset was organized to provide annotated visual data compatible with modern object detection frameworks, enabling its use in machine learning workflows involving supervised training, validation, and benchmarking. The compilation of this dataset addresses the need for domain-specific image resources suitable for developing computer vision applications applied to aquaculture monitoring, supporting research in automated counting, behavioral analysis, and intelligent production management systems.

## 3 Data Description

**Fig. 2:**
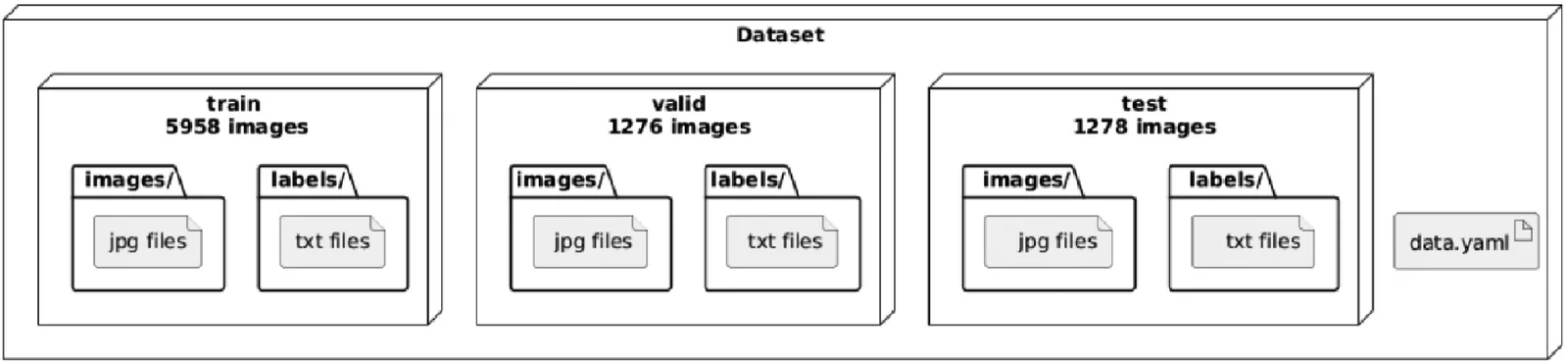
Dataset folder structure.

The dataset structure follows a hierarchical organization commonly adopted for object detection models, such as those based on the YOLO architecture (Fig. 2). It comprises 8512 samples distributed into three mutually exclusive subsets: training, validation, and testing. Each subset contains paired subdirectories storing images in JPG format and their corresponding annotation files in plain text format, preserving the direct association between visual data and bounding box labels. The dataset organization is managed through the data.yaml configuration file, which defines directory paths and class information required for model training. This structured arrangement supports reproducibility and consistent management within machine learning workflows.

**Fig. 3:**
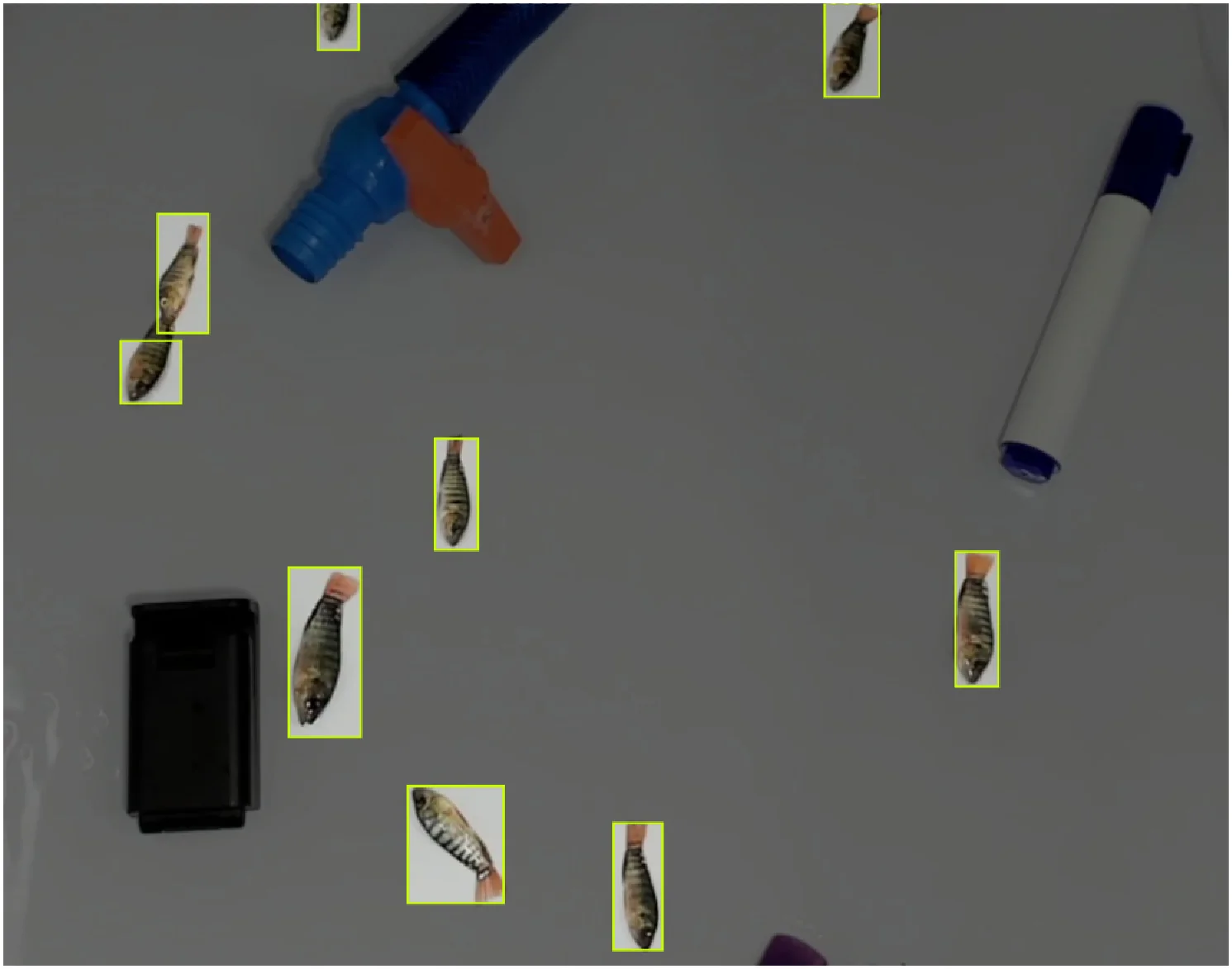
Image annotation process using the Roboflow platform, where bounding boxes are drawn around each tilapia instance and labeled according to the YOLO format.

The dataset is organized using a stratified distribution composed of training (70%), validation (15%), and testing (15%) subsets, totaling 8512 images (Table 1). The training subset contains 5958 images with 44,408 annotated instances, while the validation and testing subsets include 1276 and 1278 images, respectively, preserving similar object density across all partitions. Overall, the dataset comprises 63,004 bounding box annotations, corresponding to an average of 7.40 fish instances per image. Each image was manually annotated by drawing bounding boxes around individual tilapia specimens using the Roboflow platform, following the YOLO annotation format to maintain consistency between images and label files throughout the dataset structure. Fig. 3 presents an example image from the dataset illustrating Nile tilapia instances annotated with bounding boxes.

**Table 1:** Dataset split distribution. Number the tables consecutively in accordance with their appearance in the text.

Split	Images	Annotations (Bboxes)	% of Dataset
Train	5958	44,408	70%
Valid	1276	9013	15%
Test	1278	9583	15%
TOTAL	8512	63,004	100%

**Fig. 4:**
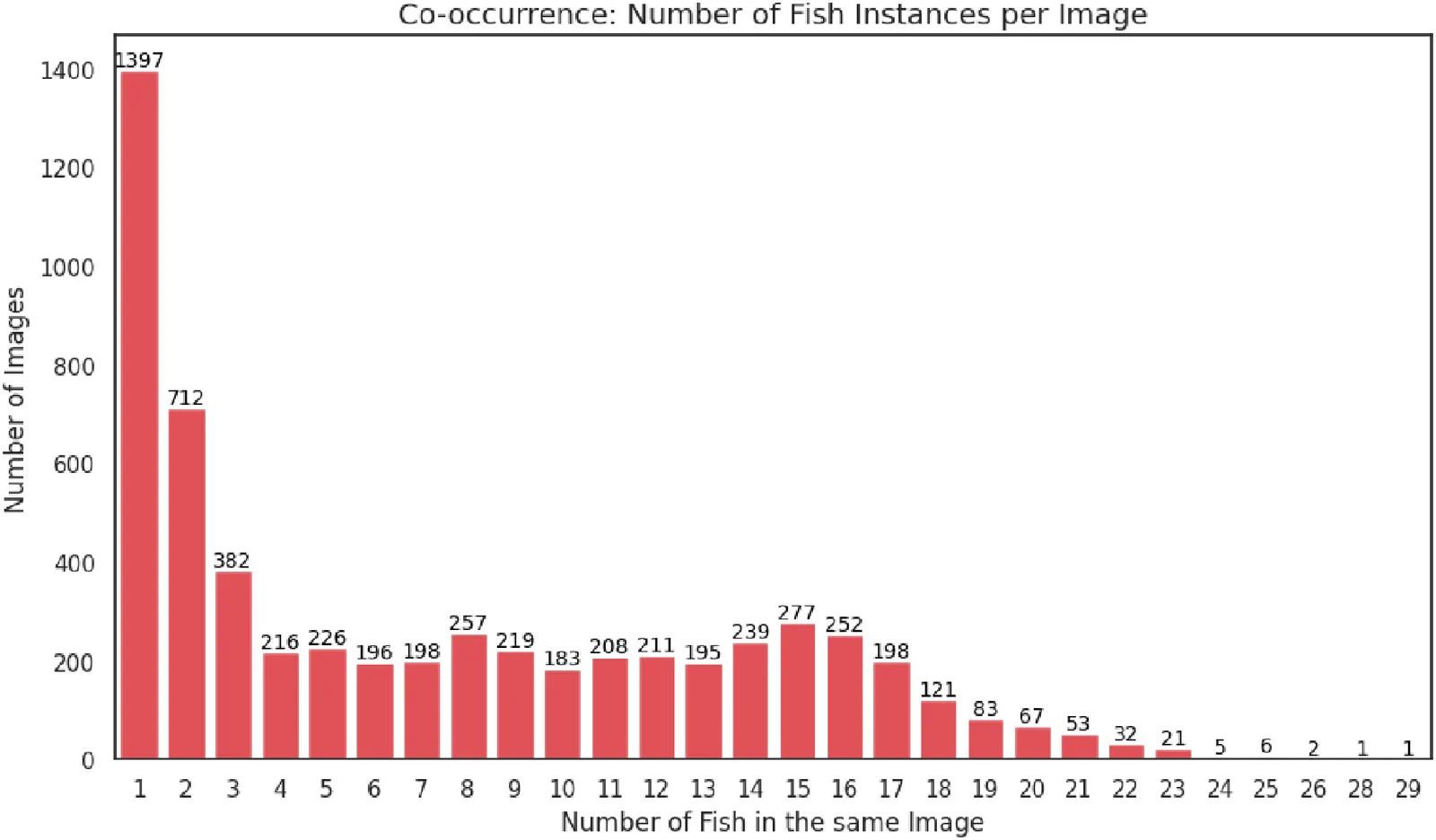
Frequency of tilapia instances per image, highlighting the demographic variability.

Analysis of the frequency of instances per image in the dataset (Fig. 4) shows high variability in the number of fish present per frame, resulting in a multimodal distribution. Although a considerable portion of the images contains isolated individuals, with 1397 images presenting only one tilapia, the dataset also includes scenes with multiple co-occurring fish, reaching up to 29 instances per image. A substantial number of samples exhibit intermediate population densities ranging from 8 to 17 fish, reflecting environments with frequent group interactions. This variation in fish quantity contributes to dataset diversity by representing both low-density and high-density observation scenarios. Images containing single individuals provide clear visual representations, while densely populated scenes include partial occlusion and overlapping fish, increasing visual complexity. Table 2 summarizes the main characteristics of the TilapiaVisionDataset, presenting an overview of its overall structure and content.

**Table 2:** Descriptive statistics of the geometric characteristics of the annotated fish instances, including bounding-box width and height, aspect ratio, and area. Values represent the distribution of 44,411 annotated instances in the dataset.

Stats	Width	Height	Aspect Ratio	Area
**Count**	44,411	44,411	44,411	44,411
**Mean**	38.10	41.91	1.13	2220.08
**Std**	29.22	33.89	0.91	5315.45
**Min**	2.00	2.00	0.10	6.00
**25%**	22.42	25.00	0.68	627.00
**50%**	33.07	35.50	1.00	1117.81
**75%**	45.00	47.00	1.18	1767.57
**Max**	403.00	407.00	10.00	1,16,874.00

The variability in the number of fish per image contributes to dataset diversification by representing both simple and complex visual scenarios. Images containing a single individual provide clear morphological representations, whereas densely populated scenes include partial occlusion and overlapping fish, increasing visual complexity within the dataset. The presence of large groups reflects situations in which multiple individuals appear in close proximity, requiring precise visual distinction between instances. Table 2 summarizes the main characteristics of the TilapiaVisionDataset, providing an overview of its structure and content.

**Fig. 5:**
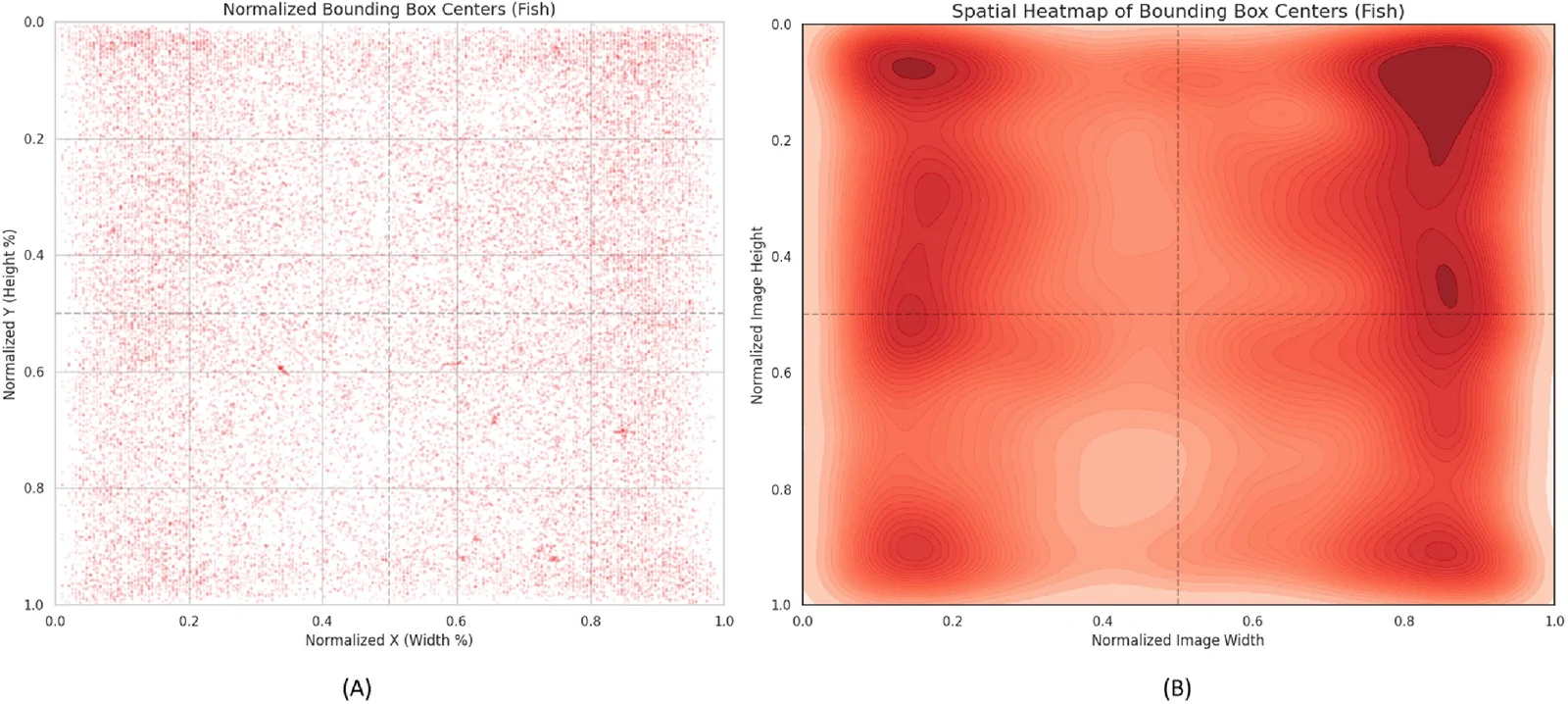
Spatial distribution bias of the instances within the flume: (A) density heatmap and (B) coordinate scatter plot.

**Fig. 6:**
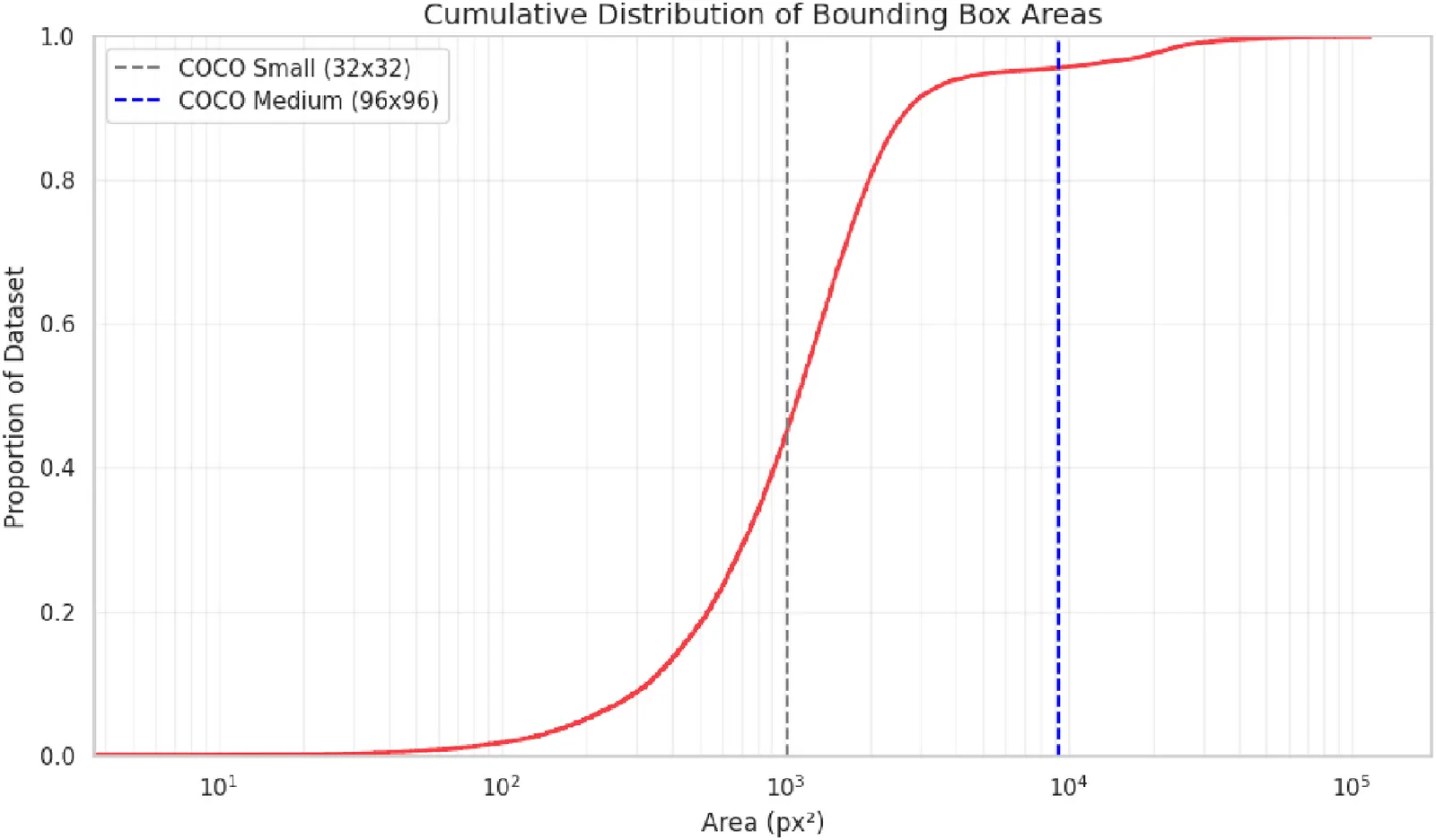
Cumulative Distribution Function (CDF) of bounding box areas. The dashed line indicates the threshold for small objects (32 × 32 pixels).

The heatmap presented in Fig. 5 illustrates the spatial distribution of bounding box centers representing tilapia instances within the images, using normalized coordinates ranging from 0 to 1. The visualization shows that fish detections are not uniformly distributed across the image area. A higher concentration of instances is observed near the edges and corners of the frames, reflecting the natural movement behavior of live fish during image acquisition, where individuals tended to swim close to the walls of the passage flume. The upper and lower corners present the highest occurrence frequencies. The heatmap provides a visual representation of the positional distribution of annotated instances within the dataset (Fig. 6).

**Fig. 7:**
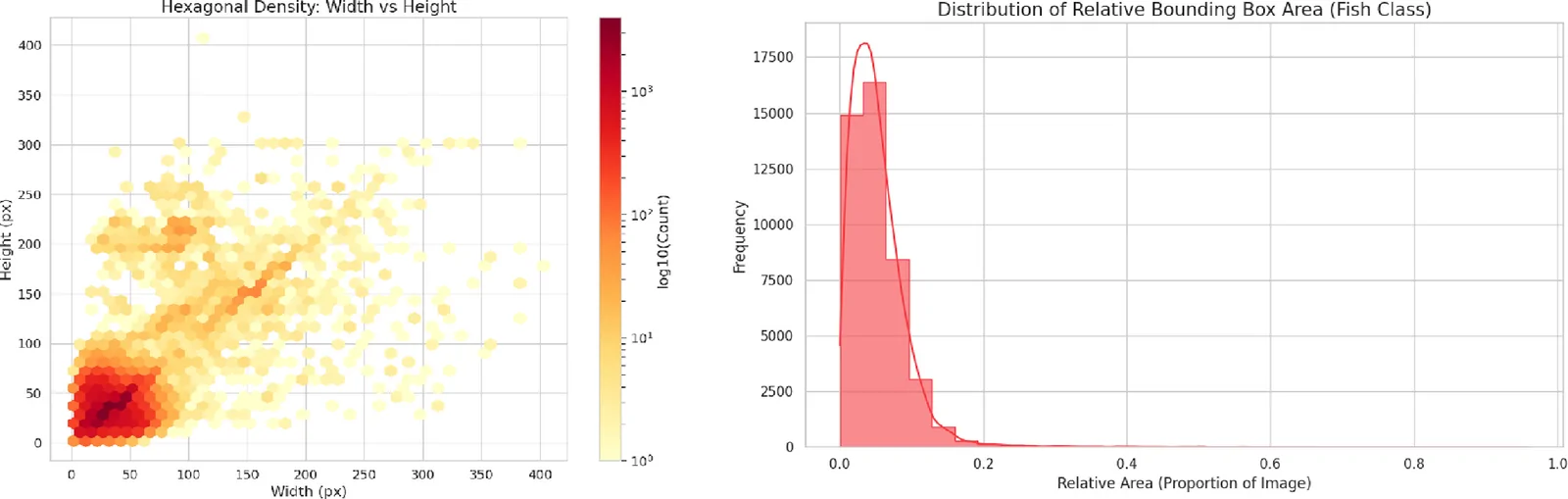
Hexagonal density plot and relative distribution of bounding boxes, correlating width and height dimensions.

The hexagonal density plot presented in Fig. 7 provides a detailed examination of the physical dimensions of the tilapia instances by correlating the width and height of each detection. Using a thermal and logarithmic color scale, this plot aggregates thousands of data points to reveal the distribution of fish sizes as well as the predominant body proportions observed in the images. The highest density region indicates that most instances correspond to small objects, with dimensions typically ranging between 20 and 50 pixels.

A diagonal distribution pattern extending from the origin can be observed, suggesting a consistent proportional relationship between object height and width as fish size increases within the images. A wider horizontal dispersion is also visible, indicating that larger tilapia instances tend to appear wider than tall, which is consistent with lateral swimming orientation relative to the camera during image acquisition.

From a geometric perspective, in Table 3, the aspect ratio presents a median value of 1.00 and a mean of 1.13, indicating that most detections approximate a square shape. However, the maximum value of 10.00 reflects the presence of partial occlusions or specific viewing angles in which the fish body appears elongated. In general, 75% of the annotated instances have widths below 45 pixels, indicating that the dataset predominantly contains small and medium-sized objects.

**Table 3:** Basic statistics of the annotated instances.

Stats	Width	Height	Aspect Ratio	Area
Count	44,41	44,41	44,41	44,41
Mean	38.10	41.91	1.13	2220.08
Std	29.22	33.89	0.91	5315.45
Min	2.00	2.00	0.10	6.00
25%	22.42	25.00	0.68	627.00
50%	33.07	35.50	1.00	1117.81
75%	45.00	47.00	1.18	1767.57
Max	403.00	407.00	10.00	116,874.00

### 3.1 Baseline validation of dataset usability

These experiments are intended solely to demonstrate dataset usability and do not represent an optimized benchmarking study. The data presented in the Table 4 comprises a comparative performance analysis among different convolutional neural network (CNN) architectures applied to the object detection task. The dataset details fundamental evaluation metrics, including F1-score, mAP@0.5 (Mean Average Precision), Precision, and Recall, covering one-stage models from the YOLO family (versions v9, v10, and v11) [4] to two-stage variants based on Faster R-CNN [5] with various backbones (ResNet, ConvNext, and RegNet). These data provide a quantitative basis to evaluate the trade-off between architectural complexity and predictive efficacy, serving as a benchmark for model selection in computer vision applications that require high accuracy and inference efficiency.

**Table 4:** Performance metrics comparison between YOLO and Faster R-CNN architectures.

Model	F1-score	mAP@0.5	Precision	Recall
YOLOv9	0.93	0.94	0.94	0.93
YOLOv10	0.92	0.94	0.94	0.92
YOLOv11	0.93	0.94	0.92	0.93
Faster R-CNN ResNet101	0.87	0.85	0.86	0.87
Faster R-CNN Nano	0.85	0.85	0.86	0.85
Faster R-CNN ConvNext Small	0.85	0.89	0.81	0.89
Faster R-CNN ConvNext Tiny	0.81	0.88	0.75	0.88
Faster R-CNN ResNet18	0.80	0.83	0.76	0.84
Faster R-CNN RegNet Y-400mf	0.79	0.82	0.74	0.84

The quantitative evaluation demonstrates that the YOLO family architectures (v9, v10, and v11) consistently outperform the Faster R-CNN variants in identifying Oreochromis niloticus specimens. The YOLOv11 model achieved superior performance in terms of mAP@0.5 (0.94), indicating high average precision in locating tilapia under test conditions. This result is particularly relevant for real-time monitoring in aquaculture, where the processing speed of one-stage models translates into an immediate response for automated feeding systems or population counting in cage-net systems.

A robust balance between precision (0.92) and recall (0.93) is observed in the YOLOv11 model, resulting in an F1-score of 0.93. In intensive fish farming scenarios, where water turbidity and fish overlapping are constant challenges, the high recall rate suggests that the model is effective in minimizing false negatives—avoiding the omission of fish present in the scene. In contrast, Faster R-CNN models, despite being architecturally more complex, showed significant performance drops, with the Faster R-CNN RegNet Y-400mf reaching an F1-score of only 0.79.

The performance discrepancy among feature extraction backbones, such as ConvNext and ResNet, underscores the importance of architectural choice for the specific morphology of Oreochromis niloticus. While Faster R-CNN ConvNext Small reached a recall of 0.89, its precision was only 0.81, suggesting a tendency to generate false alarms (false positives) compared to the stability of the YOLO series. These data provide a solid foundation for selecting computer vision algorithms in decision support systems, prioritizing models that offer not only high accuracy but also statistical reliability for biomass estimation and sanitary management of tilapia.

## 4 Experimental Design, Materials and Methods

**Fig. 8:**
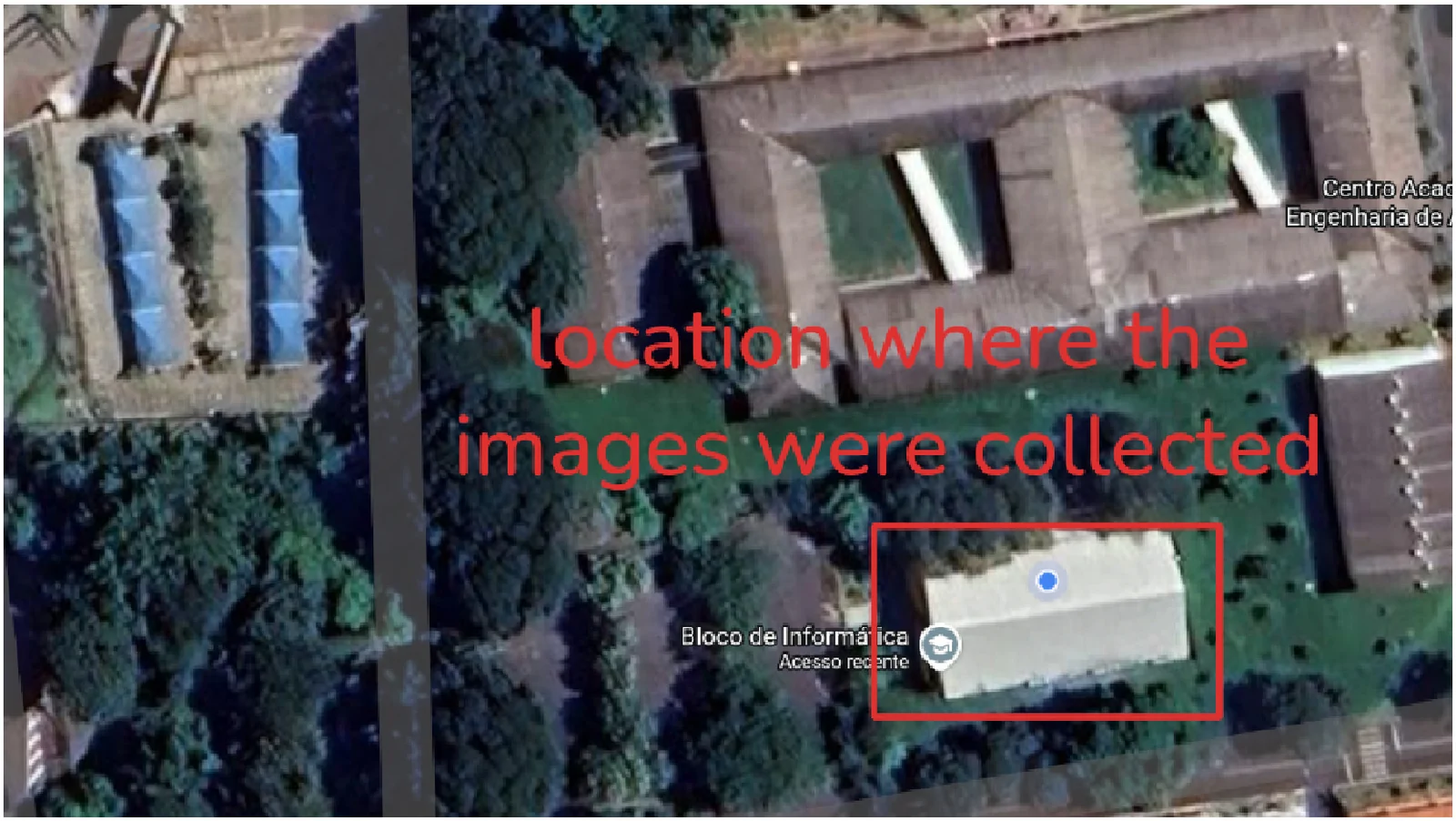
Image collection site at IF Goiano, Campus Rio Verde.

Image collection was conducted at the Artificial Intelligence Laboratory located in the Informatics Block of the Instituto Federal Goiano, Campus Rio Verde (Latitude: −17.8311, Longitude: −50.9408). The collection site is illustrated in Fig. 8.

**Fig. 9:**
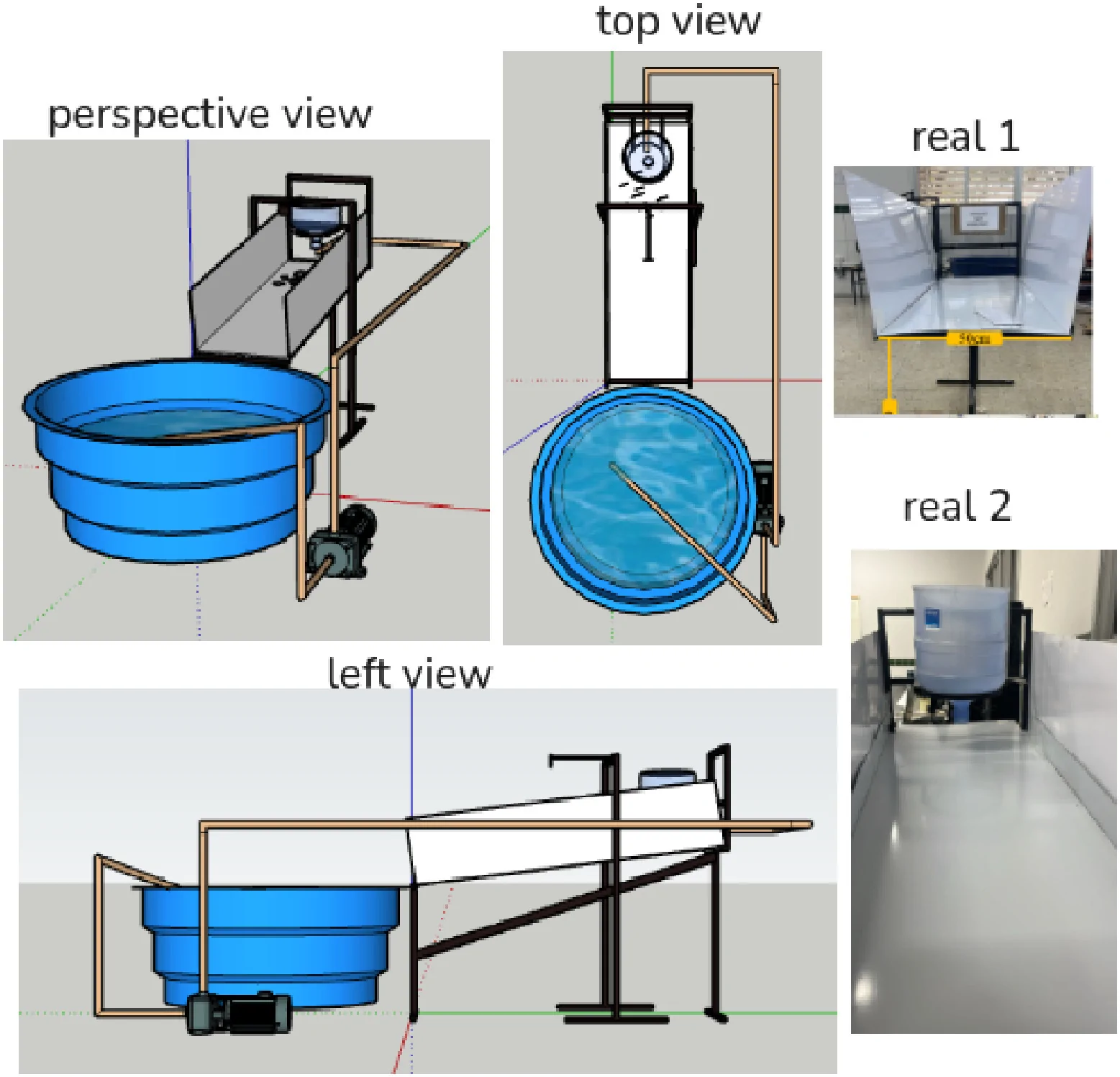
General overview of the system's test environment and experimental flume.

Fish image acquisition was performed using a passage flume integrated with a water pump system capable of maintaining a controlled water flow rate of 2700 L/h, as shown in Fig. 9. The flume dimensions are 150 cm in length, 50 cm in width, and 37 cm in wall height. The lower section includes a dedicated support designed for positioning analysis systems or auxiliary equipment, ensuring modularity, portability, and structural stability. The animal receiving structure positioned above the flume has a fitting radius of 12 cm and lateral supports measuring 18 cm.

A specialized mounting stand (trestle) was designed for camera installation. The structure is positioned 1.20 m from the ground and 40 cm above the flume. The stand has a width of 59 cm, with a 50 cm support base composed of horizontal feet and vertical rods to ensure structural stability. At the upper section, a 73 cm arm supports the camera mount at its extremity.

**Fig. 10:**
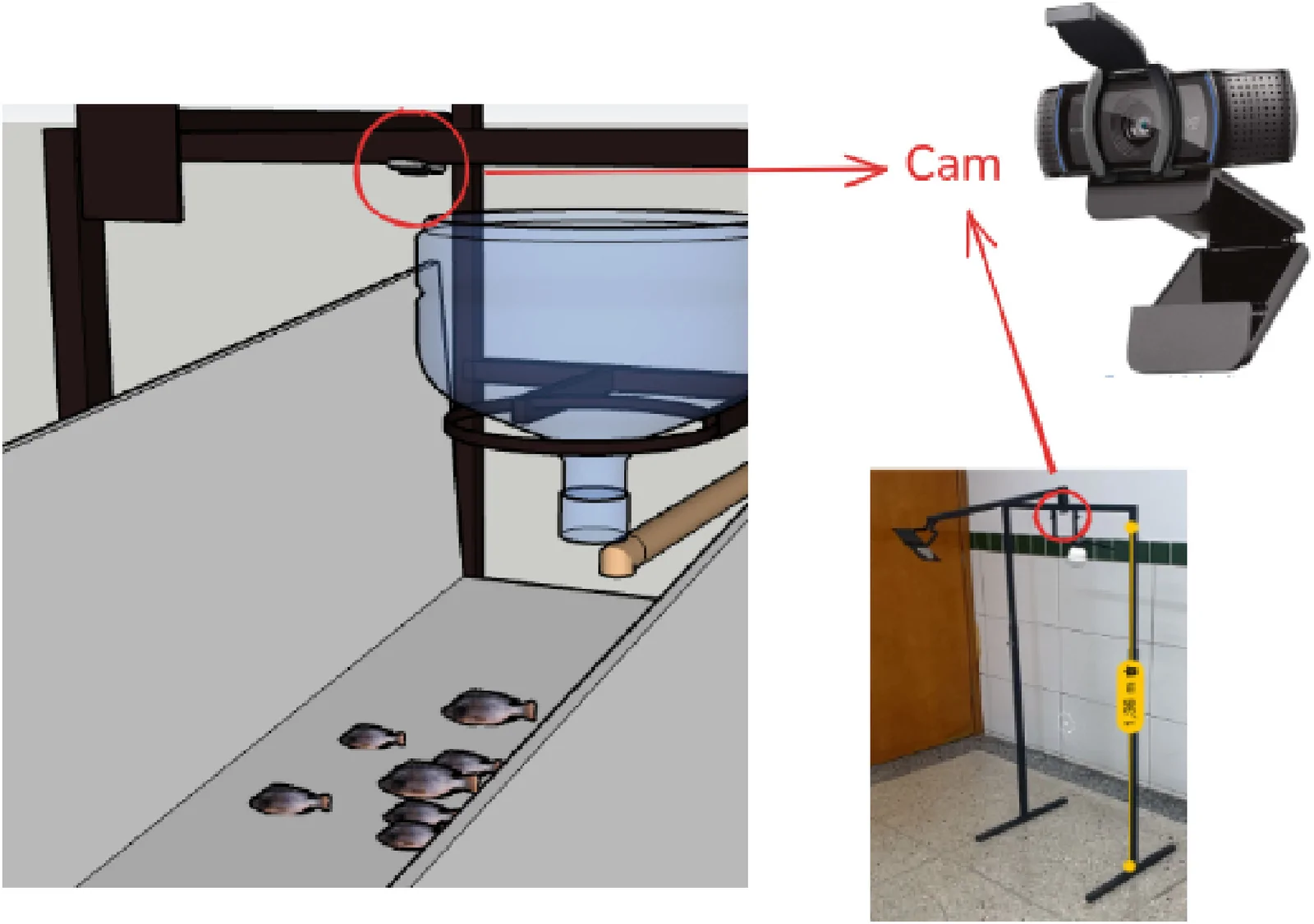
Web cam used for image capture.

For image acquisition, a Logitech C920 Full HD camera was used (Fig. 10). The device provides a maximum resolution of 1920 × 1080 pixels at 30 frames per second (fps), featuring a glass lens and a 78° field of view. The camera was installed on the mounting stand at a distance of 1.20 m from the ground and 40 cm above the flume, directly positioned over the fish flow region during data acquisition.

The handling procedures required for image acquisition were conducted using live animals. Benzocaine was applied to each fish as a sedative agent, with concentrations ranging from approximately 35 mg/L to 100 mg/L according to fish weight. The procedure followed established technical management protocols and animal welfare practices.

## Limitations

The dataset contains images of tilapia fingerlings collected under controlled conditions, which may limit the generalizability to different aquaculture environments or lighting conditions.. Some images may include occlusions or overlapping fish, which could complicate automated detection. Additionally, the dataset primarily represents healthy specimens and does not include rare phenotypes or disease conditions, limiting its applicability for studies involving abnormal or stressed fish.

## Ethics Statement

All procedures involving animals were conducted in accordance with relevant ethical guidelines. The tilapia fingerlings used for image collection were handled following standard aquaculture practices and no invasive or harmful procedures were performed. The experiments comply with the ARRIVE guidelines and with the principles of animal care recommended by the Brazilian College of Animal Experimentation, CONCEA. The sex of the animals was not considered, as the study focused on image-based phenotyping of healthy specimens.

## Credit Author Statement

**Elias Marques de Oliveira:** Conceptualization, Methodology, Software, Data Curation, Formal Analysis, Visualization, Writing – Original Draft. **Alessa Pereira Diniz Araújo:** Methodology, Data Curation, Investigation, Writing – Review and Editing. **Brenno Muller Vitorino:** Software, Data Curation, Formal Analysis, Visualization. **Vitória de Vasconcelos Kretschmer:** Investigation, Data Curation, Writing – Review and Editing. **Gabriela Almeida Marques:** Investigation, Data Curation, Writing – Review and Editing. **Lessandro do Carmo Lima:** Investigation, Data Curation, Writing – Review and Editing. **Gidelia Araujo Ferreira de Melo:** Investigation, Data Curation, Writing – Review and Editing. **Heyde Francielle do Carmo França:** Supervision, Methodology, Writing – Review and Editing. **Sergio Souza Novak:** Methodology, Software, Formal Analysis, Visualization, Writing – Review and Editing. **Adriano Carvalho Costa:** Conceptualization, Supervision, Project Administration, Funding Acquisition, Writing – Review and Editing.
